# Increased Titin Compliance Reduced Length-Dependent Contraction and Slowed Cross-Bridge Kinetics in Skinned Myocardial Strips from *Rbm*^*20ΔRRM*^ Mice

**DOI:** 10.3389/fphys.2016.00322

**Published:** 2016-07-29

**Authors:** Hannah C. Pulcastro, Peter O. Awinda, Mei Methawasin, Henk Granzier, Wenji Dong, Bertrand C. W. Tanner

**Affiliations:** ^1^Department of Integrative Physiology and Neuroscience, Washington State UniversityPullman, WA, USA; ^2^Department of Cellular and Molecular Medicine, University of ArizonaTucson, AZ, USA; ^3^Voiland School of Chemical Engineering and Bioengineering, Washington State UniversityPullman, WA, USA

**Keywords:** cross-bridge kinetics, titin compliance, length-dependent activation, Frank-Starling relationship, cardiac muscle contraction

## Abstract

Titin is a giant protein spanning from the Z-disk to the M-band of the cardiac sarcomere. In the I-band titin acts as a molecular spring, contributing to passive mechanical characteristics of the myocardium throughout a heartbeat. RNA Binding Motif Protein 20 (RBM20) is required for normal titin splicing, and its absence or altered function leads to greater expression of a very large, more compliant N2BA titin isoform in *Rbm20* homozygous mice (*Rbm20*^Δ*RRM*^) compared to wild-type mice (WT) that almost exclusively express the stiffer N2B titin isoform. Prior studies using *Rbm20*^Δ*RRM*^ animals have shown that increased titin compliance compromises muscle ultrastructure and attenuates the Frank-Starling relationship. Although previous computational simulations of muscle contraction suggested that increasing compliance of the sarcomere slows the rate of tension development and prolongs cross-bridge attachment, none of the reported effects of *Rbm20*^Δ*RRM*^ on myocardial function have been attributed to changes in cross-bridge cycling kinetics. To test the relationship between increased sarcomere compliance and cross-bridge kinetics, we used stochastic length-perturbation analysis in Ca^2+^-activated, skinned papillary muscle strips from *Rbm20*^Δ*RRM*^ and WT mice. We found increasing titin compliance depressed maximal tension, decreased Ca^2+^-sensitivity of the tension-pCa relationship, and slowed myosin detachment rate in myocardium from *Rbm20*^Δ*RRM*^ vs. WT mice. As sarcomere length increased from 1.9 to 2.2 μm, length-dependent activation of contraction was eliminated in the *Rbm20*^Δ*RRM*^ myocardium, even though myosin MgADP release rate decreased ~20% to prolong strong cross-bridge binding at longer sarcomere length. These data suggest that increasing N2BA expression may alter cardiac performance in a length-dependent manner, showing greater deficits in tension production and slower cross-bridge kinetics at longer sarcomere length. This study also supports the idea that passive mechanical characteristics of the myocardium influence ensemble cross-bridge behavior and maintenance of tension generation throughout the sarcomere.

## Introduction

Titin is the largest protein that has been identified, spanning from the Z-disk to the M-band of the cardiac sarcomere (LeWinter et al., 2007). Acting as a molecular spring in the I-band, titin contributes to passive tension as sarcomeres are stretched and influences diastolic suction, or elastic recoil at short sarcomere lengths (Granzier and Irving, 1995; Helmes et al., 1996; Wu et al., 2000). Titin compliance is primarily dependent upon differential splicing, resulting in isoforms of different lengths (Labeit and Kolmerer, 1995; Freiburg and Gautel, 1996; Wu et al., 2000). RNA Binding Motif Protein 20 (RBM20) suppresses differential titin splicing such that wild-type mice (WT) predominantly express the stiffer N2B titin isoform and homozygous *Rbm20*^Δ*RRM*^ mice express a very large, more compliant N2BA titin isoform (Guo et al., 2013; Li et al., 2013; Methawasin et al., 2014).

Actin-myosin cross-bridge behavior is regulated by intracellular [Ca^2+^] and sarcomere length, both of which are constantly changing throughout the heartbeat (for reviews see Tobacman, 1996; Cooke, 1997; Gordon et al., 2000; Kobirumaki-Shimozawa et al., 2014). Previous studies have shown that increased N2BA expression reduces passive tension (Fukuda et al., 2003; Makarenko et al., 2004; Nagueh et al., 2004; Hanft et al., 2014) which can compromise maximal Ca^2+^-activated tension production and reduce Ca^2+^-sensitivity of the tension-pCa relationship (Fukuda et al., 2001, 2003; Hanft et al., 2014; Methawasin et al., 2014). Increased myocardial compliance in *Rbm20*^Δ*RRM*^ mice and rats also demonstrated an attenuated Frank-Starling response (Methawasin et al., 2014; Ait-Mou et al., 2016). We have recently shown that cross-bridge cycling kinetics slowed at longer sarcomere length due to slowing of MgATP binding and MgADP release (Tanner et al., 2015). This led to the hypothesis that increased sarcomeric compliance in *Rbm20*^Δ*RRM*^ hearts could affect cross-bridge cycling kinetics differently at short vs. long sarcomere lengths, which may provide an explanation for compromised myocardial function in *Rbm20*^Δ*RRM*^ vs. WT myocardium.

To test this hypothesis we measured tension-pCa relationships, and cross-bridge kinetics at 1.9 and 2.2 μm sarcomere length in skinned papillary muscle strips from WT and *Rbm20*^Δ*RRM*^ mice. We found increased titin compliance in the *Rbm20*^Δ*RRM*^ strips resulted in decreased maximal tension, depressed Ca^2+^-sensitivity of the tension-pCa relationship, and slowed MgADP release compared to WT strips at each sarcomere length. As sarcomere length increased from 1.9 to 2.2 μm sarcomere length, *Rbm20*^Δ*RRM*^ strips showed a minimal increase in maximal tension Ca^2+^-sensitivity of the tension-pCa relationship, while WT strips demonstrated a robust increase in tension and Ca^2+^-sensitivity of the tension pCa relationship. These findings suggest that titin compliance influences sarcomere-length dependent activation of contraction and cross-bridge nucleotide handling rates, influencing myocardial function more greatly at longer sarcomere length.

## Materials and methods

### Animal models

All procedures were approved by the Institutional Animal Care and Use Committee at the University of Arizona and followed the U.S. National Institute of Health's “Using Animals in Intramural Research” guidelines for animal use. All mice were adult males, 25–32 weeks old. Wild-type (WT) mice were C57BL/6 strain. As previously characterized, exons 6 and 7 were deleted from the *Rbm20* mouse gene to cause an in-frame deletion of the RNA Recognition Motif (RRM) that produced the *Rbm20*^Δ*RRM*^ genotype (Methawasin et al., 2014).

### Solutions for skinned myocardial strips

Muscle mechanics solution concentrations were formulated by solving equations describing ionic equilibria according to Godt and Lindley (1982), and all concentrations are listed in mM unless otherwise noted. Dissecting solution: 133.5 NaCl, 5 KCl, 1.2 NaH_2_PO_4_, 1.2 MgSO_4_, 30 2,3-butanedione monoxime (=BDM), 10 4-(2-Hydroxyethyl)piperazine-1-ethanesulfonic acid, N-(2-Hydroxyethyl)piperazine-N′-(2-ethanesulfonic acid; = HEPES; Methawasin et al., 2014). Skinning solution: 40 N,N-Bis(2-hydroxyethyl)-2-aminoethanesulfonic acid, N,N-Bis(2-hydroxyethyl)taurine (=BES), 10 Ethylene glycol-bis(2-aminoethylether)-N,N,N′,N′-tetraacetic acid (=EGTA), 6.56 MgCl_2_, 5.88 ATP, 1 1,4-dithiothreitol (=DTT), 46.35 K propionate, 15 phosphocreatine, 0.4 Leupeptin, 0.1 trans-Epoxysuccinyl-L-leucylamido(4-guanidino)butane (=E-64), 0.5 Phenylmethanesulfonyl fluoride (=PMSF), 1% Triton X-100, pH 7.0 (Methawasin et al., 2014). Storage solution: 50 BES, 30.83 K propionate, 10 Na-azide, 20 EGTA, 6.29 ATP, 1 DTT, 20 BDM, 50 μM Leupeptin, 275 μM Pefabloc, and 1 μM E-64 with 50% glycerol wt/vol. Relaxing solution: pCa 8.0, 5 EGTA, 5 MgATP, 1 Mg^2+^, 0.3 P_i_, 20 BES, 35 phosphocreatine, 300 U/mL creatine kinase, 200 ionic strength adjusted with Na methanesulfonate, pH 7.0. Adding 0.3 mM P_i_ matches estimates for cardiac muscle (Wu et al., 2008; Weiss et al., 2015), though others use higher [Pi] (Wang et al., 2014). Activating solution: Same as relaxing with pCa 4.8. Rigor solution: same as activating solution without MgATP.

### Skinned myocardial strips

Left ventricular papillary muscles were dissected from the hearts of four WT mice and four *Rbm20*^Δ*RRM*^ mice (~180 μm in diameter and 700 μm long). Muscle strips were skinned in skinning solution overnight at 4°C, and stored at −20°C in storage solution for up to 1 week. Aluminum T-clips were attached to the end of each strip and strips were mounted between a piezoelectric motor (P841.40, Physik Instrumente, Auburn, MA) and a strain gauge (AE801, Kronex, Walnut Creek, CA), lowered into a 30 μL droplet of relaxing solution maintained at 17°C, and stretched to 1.9 or 2.2 μm sarcomere length measured by digital Fourier Transform (IonOptix Corp, Milton, MA).

### Dynamic mechanical analysis

Stochastic length perturbations were applied for a period of 60 s as previously described (Tanner et al., 2011, 2015), using an amplitude distribution with a standard deviation of 0.05% muscle lengths over the frequency range 0.5–250 Hz. Elastic and viscous moduli, *E*(ω) and *V*(ω), were measured as a function of angular frequency (ω) from the in-phase and out-of-phase portions of the tension response to the stochastic length perturbation. The complex modulus, *Y*(ω), was defined as *E*(ω) + *iV*(ω), where *i* = √−1. Fitting Equation 1 to the entire frequency range of moduli values provided estimates of six model parameters (*A, k, B, 2*π*b, C, 2*π*c*).

(1) $$\begin{array}{l} Y\left(\omega \right)\;=\;A{\left(i\omega \right)}^{k}-B\left(\frac{i\omega }{2\pi b+i\omega }\right)+C\left(\frac{i\omega }{2\pi c+i\omega }\right). \end{array}$$

The A-term in Equation (1) reflects the viscoelastic mechanical response of passive, structural elements in the muscle and holds no enzymatic dependence. The parameter *A* represents the combined mechanical stress of the fiber, while the parameter *k* describes the viscoelasticity of these passive elements, where *k* = 0 represents a purely elastic response and *k* = 1 is a purely viscous response (Mulieri et al., 2002; Palmer et al., 2013). The B- and C-terms in Equation (1) reflect enzymatic cross-bridge cycling behavior that produce frequency-dependent shifts in the viscoelastic mechanical response during Ca^2+^-activated contraction. These B- and C-processes characterize work-producing (cross-bridge attachment or recruitment) and work-absorbing (cross-bridge detachment) muscle responses, respectively (Kawai and Halvorson, 1991; Zhao and Kawai, 1993; Campbell et al., 2004; Palmer et al., 2007). The parameters *B* and *C* represent the mechanical stress from the cross-bridges (i.e., number of cross-bridges formed × their mean stiffness), and the rate parameters 2π*b* and 2π*c* reflect cross-bridge kinetics that are sensitive to biochemical perturbations affecting enzymatic activity, such as [MgATP], [MgADP], or [P_i_] (Lymn and Taylor, 1971). Molecular processes contributing to cross-bridge attachment or tension generation underlie the cross-bridge attachment rate, 2π*b*. Similarly, processes contributing to cross-bridge detachment or tension decay underlie the cross-bridge detachment rate, 2π*c*.

Stochastic system analysis provides a portrait of cross-bridge kinetics as a function of [MgATP]. Assuming that the myosin attachment events include time spent in the MgADP state and in the rigor state, the cross-bridge detachment rate can be described by:

(2) $$\begin{array}{l} 2\pi c=\frac{k_{-ADP}\left[MgATP\right]}{\frac{k_{-ADP}}{k_{+ATP}}+\left[MgATP\right]}. \end{array}$$

As explained in detail by Tyska and Warshaw (2002) and implemented in our previous publications (Wang et al., 2013; Tanner et al., 2015), fitting the 2π*c*-[MgATP] relationship to Equation 2 allows a calculation of (i) *k*_−*ADP*_, which represents cross-bridge MgADP release rate and the asymptotic, maximal myosin detachment rate in s^−1^ at saturating [MgATP]; and (ii) *k*_+*ATP*_, which represents the second-order cross-bridge MgATP binding rate per myosin concentration in M^−1^ s^−1^.

### Statistical analysis

All values are shown as mean ± SEM. Constrained non-linear least squares fitting of Equations (1, 2) to moduli was performed using sequential quadratic programming methods in Matlab (v 7.9.0, The Mathworks, Natick MA). All statistical tests were performed using SPSS (IBM Statistics, Chicago, IL). A two-way ANOVA was used to assess effects of genotype and sarcomere length for parameter estimates from (i) the 3-parameter Hill fits to the tension-pCa relationships and (ii) the parameter estimates from fits to Equation (2) for the nucleotide handling rates. All other relationships were analyzed using linear mixed models with pCa, frequency, or MgATP as a repeated measure, followed by a least significant difference *post-hoc* comparison of the means between genotype or sarcomere length. Statistical significance is reported at *p* < 0.05.

## Results

**Figure 1:**
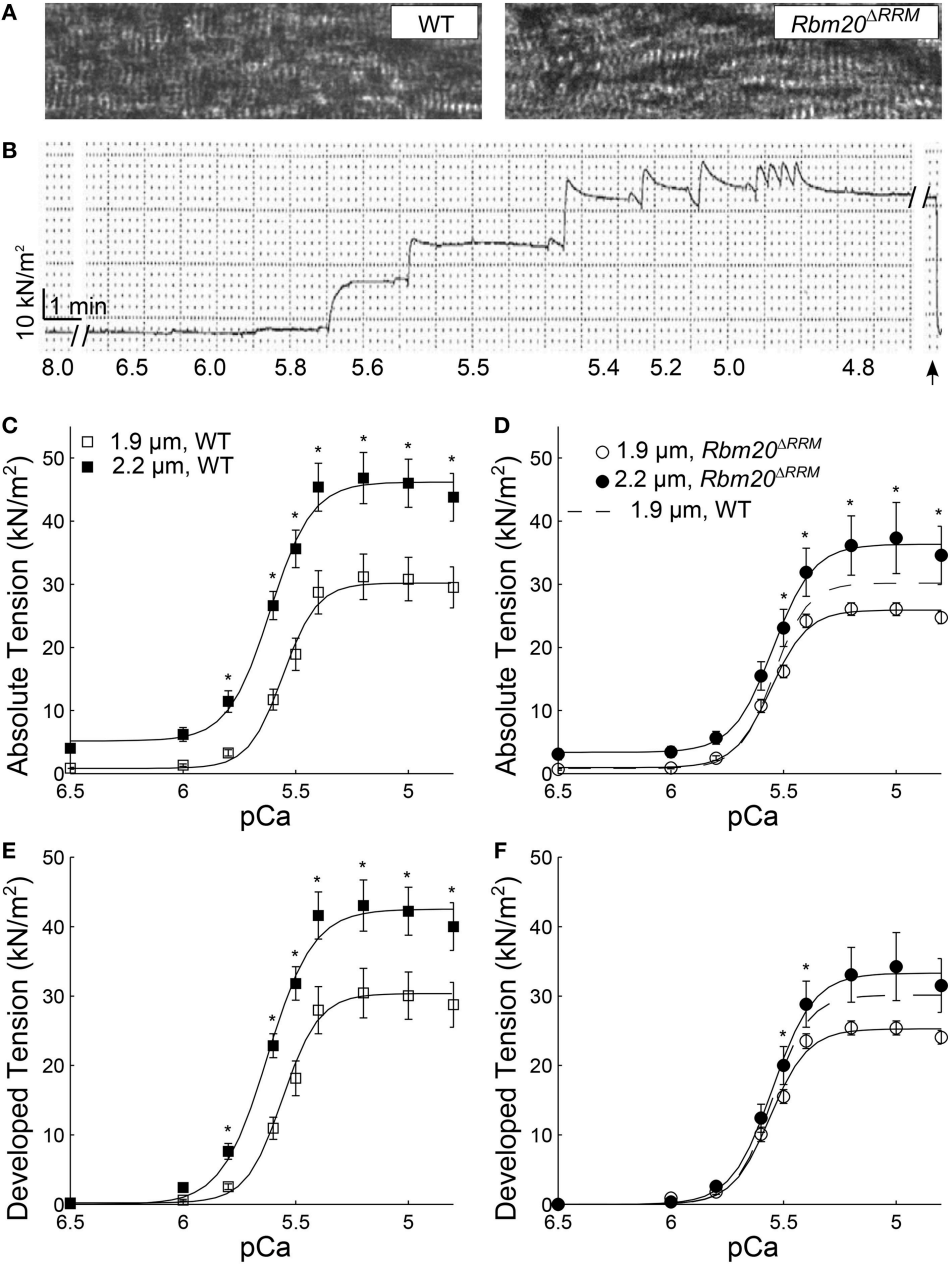
**Tension-pCa relationships at long vs. short sarcomere length. (A)** Example light microscopy images at 40X, showing skinned myocardial strips from WT (left), and *Rbm20*^Δ*RRM*^ (right) mice. **(B)** An example absolute tension trace plotted against time from a myocardial strip that was Ca^2+^-activated from pCa 8.0 to 4.8 (pCa values listed below each solution exchange), where the strip was slacked (arrow) near the end of the experiment to ensure no baseline-tension changes throughout the time course of an experiment. Absolute tension-pCa relationships for **(C)** WT, and **(D)** *Rbm20*^Δ*RRM*^ mice and developed tension-pCa relationships for **(E)** WT and **(F)** *Rbm20*^Δ*RRM*^ mice at 1.9 and 2.2 μm sarcomere length. Solid lines represent 3-parameter Hill fits to the tension-pCa data, with the dashed lines representing the 1.9 μm sarcomere length fit for *Rbm20*^Δ*RRM*^ replotted in panel **(D,F)**. ^*^p < 0.05 between sarcomere length within a genotype.

There were no obvious differences in sarcomere organization or monitored sarcomere length in skinned papillary muscle strips from WT and *Rbm20*^Δ*RRM*^ mice (Figure 1A). As skinned myocardial strips were Ca^2+^-activated from pCa 8.0 to pCa 4.8, steady-state, isometric tension developed in a sigmoidal manner that was fit to a 3-parameter Hill equation (Figures 1B–F, Table 1). These tension-pCa relationships are shown two different ways, where: (i) where absolute tension values (=measured force values normalized to cross-sectional area of each myocardial strip; Figures 1C,D) illustrate the total tension produced by the strip (i.e., both the passive tension value at pCa 8.0 plus the Ca^2+^-activated active tension values), and (ii) developed tension values illustrate the Ca^2+^-activated tension produced by the strip (i.e., absolute tension minus the passive, relaxed tension value at pCa 8.0; Figures 1E,F).

**Table 1:** **Characteristics of tension-pCa relationships in mouse myocardium at 1.9 and 2.2 μm sarcomere lengths, with and without ***Rbm20**^**ΔRRM**^* mutation (mean ± SEM)**.

	**WT 1.9 μm**	**WT 2.2 μm**	***Rbm20^ΔRRM^* 1.9 μ*m***	***Rbm20^ΔRRM^* 2.2 μ*m***
T_min_ (kN/*m*^2^)	0.75 ± 0.11	3.78 ± 0.69^*^	0.66 ± 0.11	3.07 ± 0.89^*^
T_max_ (kN/*m*^2^)	29.50 ± 3.26	43.78 ± 3.78^*^	24.72 ± 0.97	34.58 ± 4.62^*^^†^
T_dev_ (kN/m^2^)	28.75 ± 3.23	39.99 ± 3.45^*^	24.06 ± 0.99	31.51 ± 3.88
pCa_50_	5.55 ± 0.01	5.63 ± 0.02^*^	5.56 ± 0.01	5.56 ± 0.03^†^
n_H_	5.48 ± 0.30	4.48 ± 0.20^*^	5.43 ± 0.27	5.85 ± 0.28^†^
Max_fit_ (kN/m^2^)	30.16 ± 3.43	42.46 ± 3.59^*^	25.27 ± 1.02	33.42 ± 4.34^†^
n fibers	9	8	7	9

T_min_, absolute tension value at pCa 8.0.

T_max_, absolute tension value at pCa 4.8.

T_dev_, Ca^2+^-activated, developed tension (T_max_–T_min_).

Max_fit_, pCa_50_, and n_H_ represent fit parameters to a 3-parameter Hill equation for the T_dev-pCa_ relationship: $T_{dev}(pCa)=\frac{Max_{fit}}{1+10^{n_{H}(pCa-pCa_{50})}}$.

† *p < 0.05 effect of mutation at same sarcomere length*.

* p < 0.05 effect of sarcomere length within a mutation/genotype.

Under maximally activated conditions, myocardial strips with both WT and *Rbm20*^Δ*RRM*^ genotypes displayed greater absolute tension at 2.2 vs. 1.9 μm sarcomere length (Figures 1C,D). Relaxed tension values (pCa 8.0) were also greater at the longer sarcomere length in both genotypes (Table 1). Developed tension was greater at 2.2 vs. 1.9 μm sarcomere length from pCa 5.8–4.8 in myocardial strips from WT mice (Figure 1E). However, in myocardial strips from the *Rbm20*^Δ*RRM*^ mice, developed tension was only greater at 2.2 μm sarcomere length at pCa 5.5 and 5.4 (Figure 1F). Thus, Ca^2+^-sensitivity of the tension-pCa relationship increased with sarcomere length in the WT strips (by ~0.08 pCa units), but this sarcomere length-dependent increase in Ca^2+^-sensitivity of tension was lost in *Rbm20*^Δ*RRM*^ strips (Table 1). At 2.2 μm sarcomere length, WT strips also displayed greater Ca^2+^-sensitivity of tension than *Rbm20*^Δ*RRM*^ strips (by ~0.07 pCa units). In WT strips, the Hill coefficient (n_H_) for the tension-pCa relationship was smaller at 2.2 vs. 1.9 μm sarcomere length, indicating reduced cooperativity at longer sarcomere length (Table 1). At 2.2 μm sarcomere length, n_H_ was smaller for WT strips than *Rbm20*^Δ*RRM*^ strips; there were no differences in n_H_ between genotypes at 1.9 μm sarcomere length.

**Figure 2:**
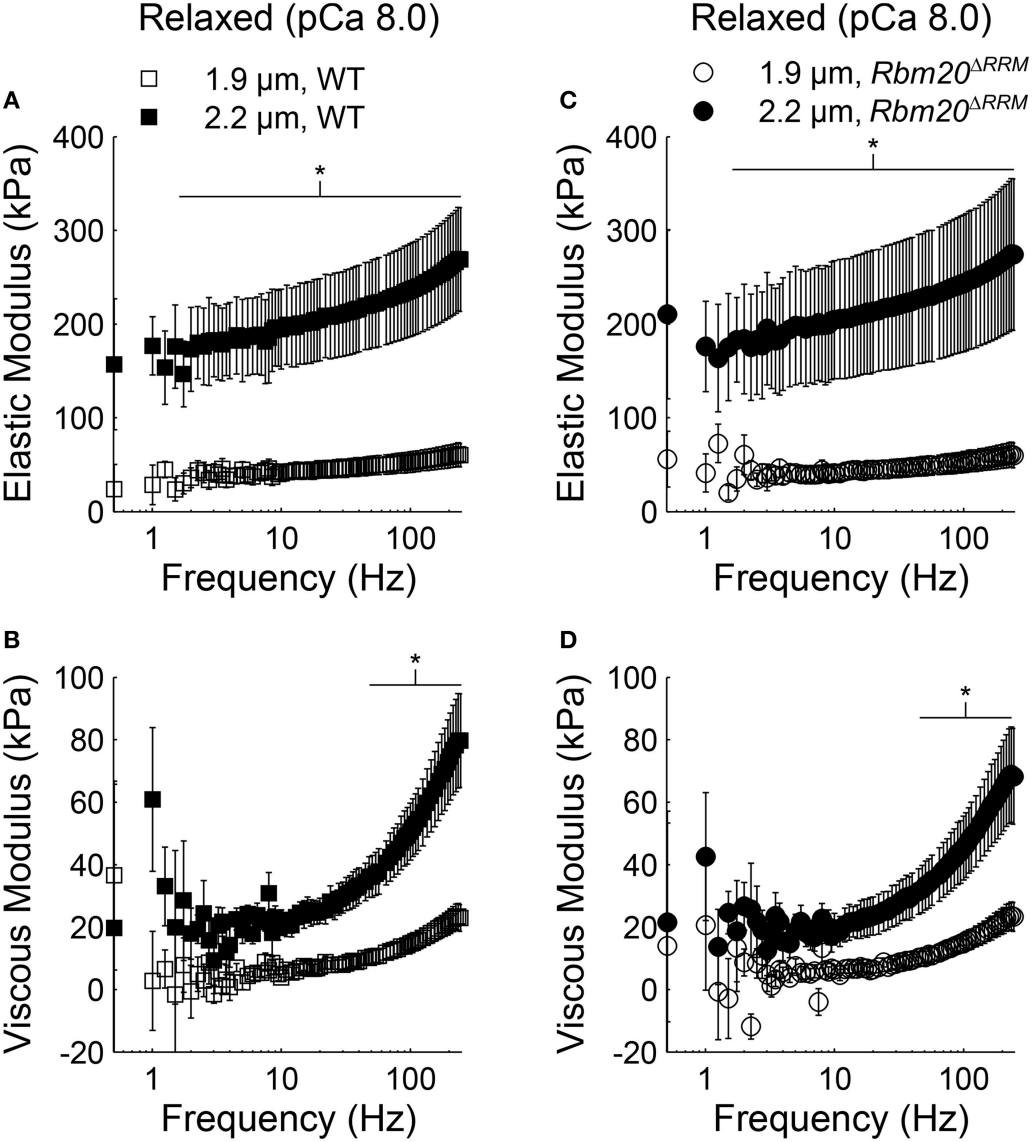
**Sarcomere length affected myocardial viscoelasticity under relaxed conditions in *Rbm20*^Δ*RRM*^ and WT**. Elastic moduli were plotted against frequency for **(A)** WT and **(C)** *Rbm20*^Δ*RRM*^ genotypes for skinned papillary muscle strips at 1.9 and 2.2 μm sarcomere lengths under relaxed conditions (pCa 8 and 5 mM MgATP). The associated viscous moduli were plotted against frequency for **(B)** WT and **(D)** *Rbm20*^Δ*RRM*^ at 1.9 and 2.2 μm sarcomere lengths. ^*^p < 0.05 between sarcomere lengths within a genotype.

**Figure 3:**
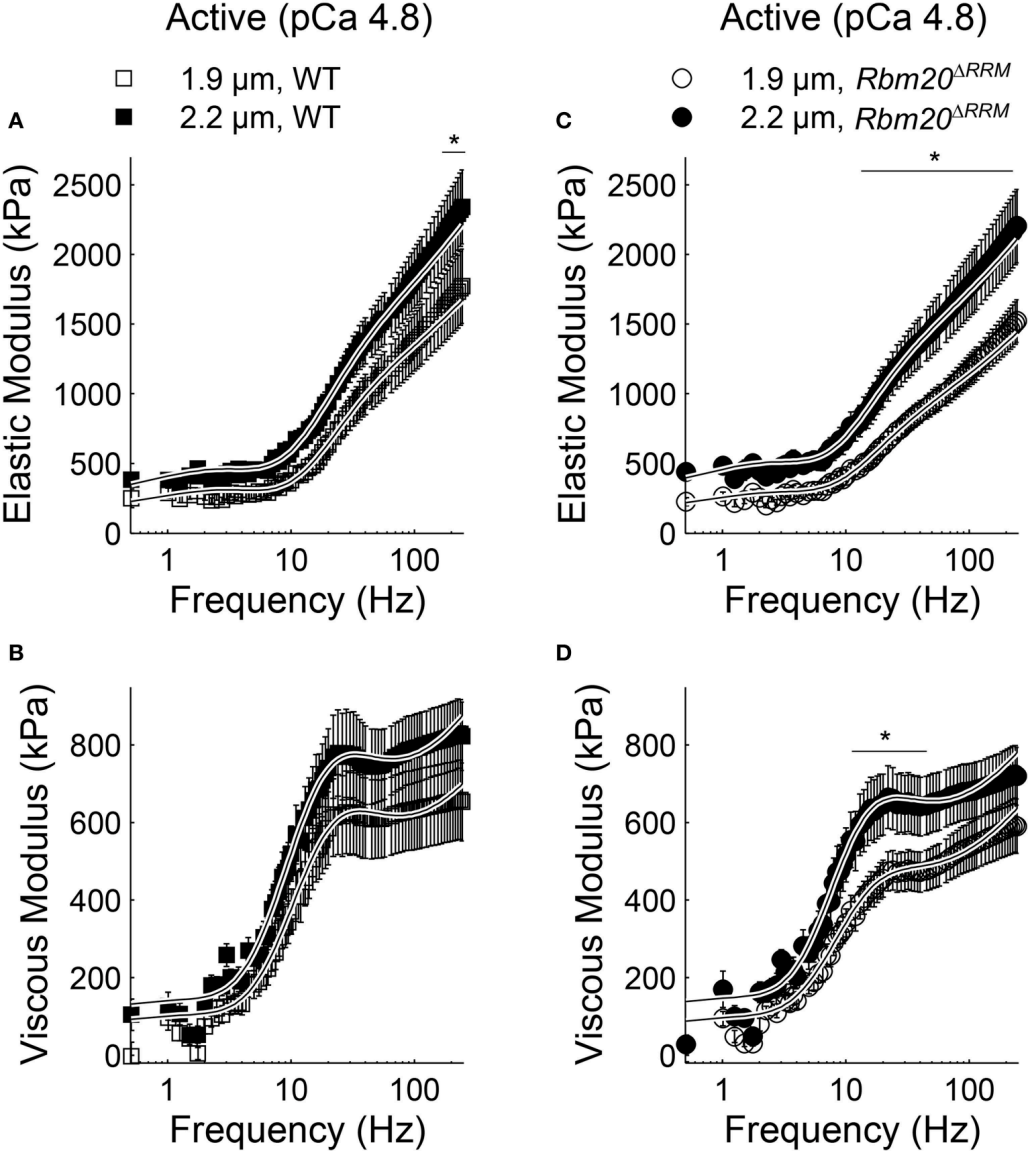
**Sarcomere length affected myocardial elasticity in *Rbm20*^Δ*RRM*^ and WT**. Elastic moduli were plotted against frequency for **(A)** WT and **(C)** *Rbm20*^Δ*RRM*^ genotypes for skinned papillary muscle strips at 1.9 and 2.2 μm sarcomere lengths under activated conditions (pCa 4.8 and 5 mM MgATP). The associated viscous moduli were plotted against frequency for **(B)** WT and **(D)** *Rbm20*^Δ*RRM*^ at 1.9 and 2.2 μm sarcomere lengths. ^*^p < 0.05 between sarcomere lengths within a genotype.

In both genotypes under relaxed conditions (pCa 8.0), elastic moduli values were greater at 2.2 vs. 1.9 μm sarcomere length for all frequencies >1.5 Hz (Figures 2A,C). Viscous moduli values were also greater at longer sarcomere length at frequencies >51 Hz in WT strips and frequencies >54 Hz in *Rbm20*^Δ*RRM*^ strips (Figures 2B,C, respectively). Under activated conditions (pCa 4.8, 5 mM MgATP), elastic moduli values were greater at longer sarcomere length for frequencies above 145 Hz in WT (Figure 3A), and frequencies >22 Hz in *Rbm20*^Δ*RRM*^ strips (Figure 3C). In addition to these moduli differences, there was a consistent shift toward lower frequencies for the overall elastic moduli-frequency relationship at longer sarcomere length; this shift toward lower frequencies was larger for *Rbm20*^Δ*RRM*^ strips vs. WT strips. Under activated conditions, viscous moduli were not different at any particular sarcomere length in the WT strips (Figure 3B), and viscous moduli were greater at 2.2 vs. 1.9 μm sarcomere length at frequencies between 9.5 and 54 Hz in the *Rbm20*^Δ*RRM*^ strips (Figure 3D). There was also a consistent shift toward lower frequencies for the overall viscous moduli-frequency relationship at longer sarcomere length; this shift toward lower frequencies was larger for *Rbm20*^Δ*RRM*^ strips vs. WT strips. Altogether these data indicate greater myocardial viscoelasticity at longer sarcomere length under relaxed and activated conditions, although the influence of titin compliance was minimal as there were no significant effects of genotype in the moduli-frequency relationships (Figures 2, 3). The length-dependent shifts toward lower frequencies in the moduli-frequency relationships at pCa 4.8 indicate slower cross-bridge cycling as sarcomere length increased for both genotypes, although this slowing was greater for *Rbm20*^Δ*RRM*^.

**Figure 4:**
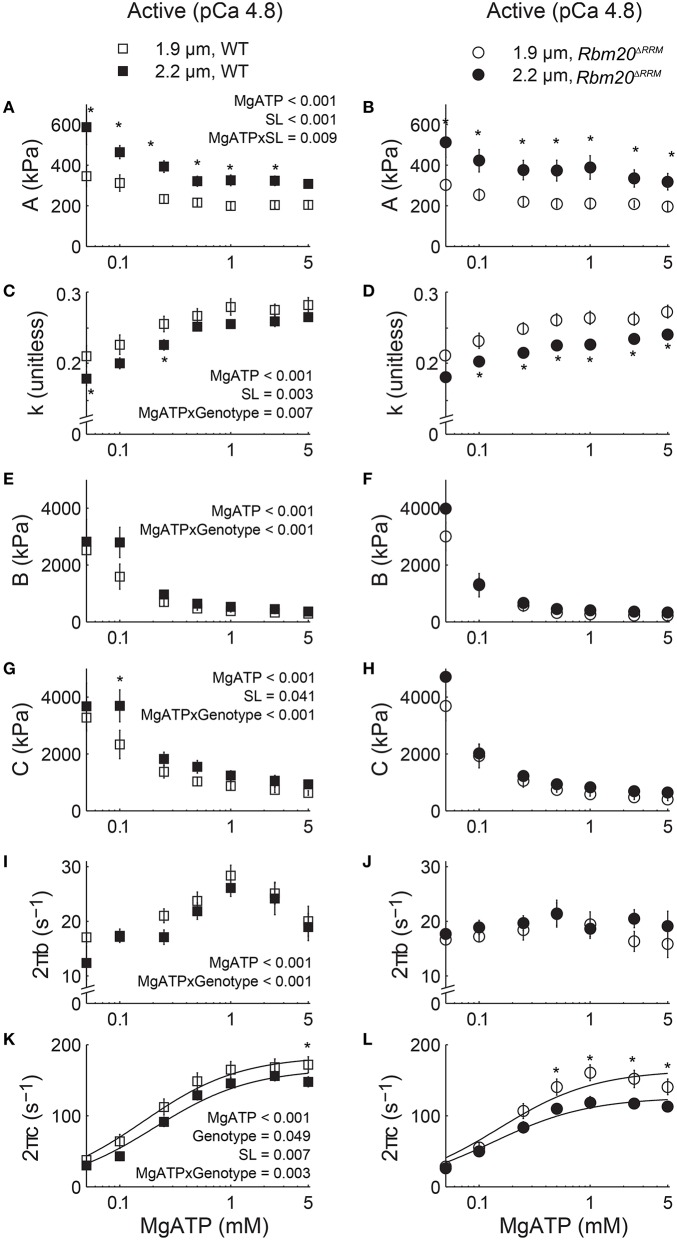
**Increased titin compliance affected myocardial viscoelasticity and cross-bridge kinetics as [MgATP] varied at pCa 4.8**. Parameter fits to Equation (1) are plotted against [MgATP] at 1.9 and 2.2 μm sarcomere lengths for skinned papillary muscle strips from WT (left set of panels) and *Rbm20*^Δ*RRM*^ (right set of panels) mice. Myocardial viscoelastic stiffness increased and became increasingly elastic as [MgATP] decreased, as reflected by the MgATP-dependent increase in *A* **(A,B)** and decrease in *k* (**C,D)**. Magnitude parameters for the B-process **(E,F)** and the C-process **(G,H)** also increased as [MgATP] decreased, which indicates an expected increase in cross-bridge binding as [MgATP] was titrated toward rigor. The rate of cross-bridge attachment, 2πb **(I,J)**, and the rate of cross-bridge detachment, 2πc **(K,L)**, decreased as [MgATP] decreased, which indicates the expected slowing of cross-bridge cycling kinetics as [MgATP] was titrated toward rigor. Dashed lines representing the *Rbm20*^Δ*RRM*^, 1.9 μm sarcomere length data were replotted in the left set of panels. *P*-values listed within the left panel show significant (<0.05) main effects of [MgATP], genotype, sarcomere length (SL), and any interactions between these effects among all four sets of data, resulting from mixed models analysis of each parameter-[MgATP] relationship. ^*^p < 0.05 between sarcomere lengths within a genotype.

Moduli values were fit to Equation (1) to extract model parameters related to viscoelasticity, cross-bridge binding, and cross-bridge kinetics as the skinned strips were titrated toward rigor (5.0–0.05 mM MgATP, pCa 4.8). These model parameters are plotted against [MgATP] in Figure 4, with *p*-values listed in the left panel for each parameter that demonstrated significant main effects or interactions from the mixed-model analysis. As [MgATP] was titrated toward rigor, *A* values increased and *k* values decreased for both genotypes, suggesting increased viscoelastic myocardial stiffness that became more elastic (vs. viscous) due to greater cross-bridge binding as MgATP decreased (Figures 4A–D). In both genotypes, *A* values were greater and *k* values were smaller at 2.2 vs. 1.9 μm sarcomere length, which represents greater myocardial viscoelasticity due to a combination of: (i) passive elements of the sarcomere being stretched or extended more at 2.2 vs. 1.9 μm sarcomere length and (ii) greater binding of slower-cycling cross-bridges at 2.2 vs. 1.9 μm sarcomere length. For both genotypes, the values for *B* and *C* increased as [MgATP] was titrated toward rigor and the magnitudes for *C* increased at 2.2 vs. 1.9 μm sarcomere length (Figures 4E,F), also suggesting greater cross-bridge binding at longer sarcomere length.

As [MgATP] decreased, cross-bridge attachment rate (2πb, Figures 4I,J) slowed in both genotypes. The significant MgATP × genotype interaction suggests cross-bridge attachment rate was more sensitive to [MgATP] in WT than in *Rbm20*^Δ*RRM*^ strips, although cross-bridge attachment rates were not different at 2.2 vs. 1.9 μm sarcomere length. Similarly, as [MgATP] decreased toward rigor, cross-bridge detachment rate (2πc, Figures 4K,L) slowed in both genotypes. Cross-bridge detachment rates were also slower at 2.2 vs. 1.9 μm sarcomere length for both genotypes. The significant genotype effect on cross-bridge detachment rate suggests that *Rbm20*^Δ*RRM*^ strips displayed a slower cross-bridge detachment rate than WT strips across the entire [MgATP] range, although this statistic was primarily driven by the slowest detachment rates occurring for *Rbm20*^Δ*RRM*^ strips at 2.2 μm sarcomere length. Again the significant MgATP × genotype interaction suggests that cross-bridge detachment rate was more sensitive to [MgATP] in WT than in *Rbm20*^Δ*RRM*^ strips.

Fitting the 2πc-MgATP relationship to Equation (2) (solid lines in Figures 4K,L) provides an estimate of the cross-bridge rates of MgADP release (*k*_−*ADP*_) and MgATP binding (*k*_+*ATP*_). The MgADP release rate slowed with increased titin compliance for the *Rbm20*^Δ*RRM*^ fibers, and there was a length-dependent slowing of *k*_−*ADP*_ at longer sarcomere length for both genotypes (Table 2). For WT fibers, increasing sarcomere length from 1.9 to 2.2 μm slowed MgADP release by 12% (*p* = 0.015 using a *t*-test). For *Rbm20*^Δ*RRM*^ fibers *k*_−*ADP*_ slowed 22% as sarcomere length increased from 1.9 to 2.2 μm, showing about twice as much length-dependent slowing of *k*_−*ADP*_ for *Rbm20*^Δ*RRM*^ than WT. However, increased titin compliance in the *Rbm20*^Δ*RRM*^ strips led to slower rates of MgADP release at both sarcomere lengths (13 and 23% slower at short and long sarcomere length, respectively), compared to WT *k*_−*ADP*_ values. The cross-bridge rate of MgATP binding did not differ with genotype or with sarcomere length (Table 2). These finding suggest that increased compliance of the myofilament lattice slows cross-bridge cycling kinetics, primarily due to slower MgADP dissociation from cross-bridges.

**Table 2:** **Estimates of myosin cross-bridge kinetics from fits of the cross-bridge detachment rate (2πc) vs. MgATP relationships to Equation (2) for 1.9 and 2.2 μm sarcomere lengths (mean ± SEM)**.

	**WT 1.9 μm**	**WT 2.2 μm**	***Rbm20^ΔRRM^* 1.9 μ*m***	***Rbm20^ΔRRM^* 2.2 μ*m***
k_−ADP_ (s^−1^)	189.64 ± 12.42	167.83 ± 7.16	164.41 ± 12.27^‡^	128.75 ± 7.33^†^^*^
k_+ATP_ (mM^−1^ s^−1^)	1225.46 ± 193.46	842.04 ± 123.64	1132.56 ± 134.14	988.29 ± 177.37

*k_-ADP_, cross-bridge MgADP release rate*.

*k_+ATP_, cross-bridge MgATP binding rate*.

† *p < 0.05*,

‡ *p < 0.1 effect of mutation at same sarcomere lengths*.

* *p < 0.05 effect of sarcomere length under similar treatment conditions*.

## Discussion

Computational simulations of muscle contraction have demonstrated that mechanical characteristics of the sarcomere (i.e., filament, cross-bridge, and titin compliance; compliance = stiffness^−1^) influence the dynamics of cross-bridge binding and tension generation in a muscle fiber (Daniel et al., 1998; Martyn et al., 2002; Chase et al., 2004; Campbell, 2006, 2009, 2016; Sheikh et al., 2012; Tanner et al., 2012a, 2014). These mathematical models predict that increasing sarcomeric compliance diminishes steady-state tension, slows the apparent rate of tension development, slows cross-bridge cycling rates, and can impact the rate of tension relaxation as well. As RMB20^Δ*RRM*^ mice express more of the compliant N2BA titin isoform than the WT (Guo et al., 2013; Methawasin et al., 2014), these transgenic animals represent a useful model system to test some of these model predictions and directly assess the role of titin compliance in length-dependent tension production and ensemble cross-bridge behavior in skinned myocardial strips. In this study we observed that increased titin compliance in *Rbm20*^Δ*RRM*^ fibers diminished steady-state tension, reduced Ca^2+^-sensitivity of the tension-pCa relationship, and slowed cross-bridge detachment rate due to slowed MgADP dissociation from strongly-bound cross-bridges. The effects of titin compliance were sarcomere length-dependent, showing almost no length-dependent tension response in *Rbm20*^Δ*RRM*^ strips, in contrast to the robust length-dependent increase in maximal tension and Ca^2+^-sensitivity of the tension-pCa relationships between 1.9 and 2.2 μm sarcomere length in WT strips. This length-dependent activation response was eliminated in *Rbm20*^Δ*RRM*^ strips despite a slowed cross-bridge detachment rate as sarcomere length increased, which would be expected to enhance thin-filament activation at 2.2 μm sarcomere length due to strong cross-bridge binding (Bremel and Weber, 1972; Wang and Fuchs, 1994; Metzger, 1995; Fitzsimons and Moss, 1998; Smith et al., 2009; Terui et al., 2010; Li et al., 2014). Empirical findings in this study support previous computational simulations predicting the important role that sarcomeric compliance plays in muscle contraction and further suggests that titin mechanics affect length dependent activation of contraction, perhaps by altering how tension propagates throughout the sarcomere to influence thin-filament activation.

Our observations that increased titin compliance in the *Rbm20*^Δ*RRM*^ strips reduced maximal tension values and decreased Ca^2+^ sensitivity of tension agree with previous findings that suggest greater N2BA titin isoform expression depresses maximum tension production (Makarenko et al., 2004; Lewinter et al., 2010; Patel et al., 2012; Hanft et al., 2014; Methawasin et al., 2014). Our measurements also show that effects of titin compliance on Ca^2+^-activated tension are sarcomere length-dependent, supporting previous studies showing that increased titin compliance depresses tension more significantly at longer sarcomere length (Fukuda et al., 2003; Methawasin et al., 2014). This is most evident by the similar tension-pCa relationships at 1.9 μm sarcomere length among both genotypes (Figure 1; Table 1), with a robust length-dependent increase in Ca^2+^-activated tension production as sarcomere length increased to 2.2 um for WT strips that did not occur for *Rbm20*^Δ*RRM*^ strips. These data suggest that length-dependent activation of contraction and the slope of the ascending limb of the sarcomere-length vs. Ca^2+^-activated tension relationship may depend upon mechanical characteristics of titin. This implies that dynamic processes related to cross-bridge cycling kinetics, thin-filament activation, and tension development within the sarcomere may be influenced by the mechanical characteristics of titin.

Cross-bridge detachment rates slowed as sarcomere length increased from 1.9 to 2.2 μm among both genotypes, but the slowing was more pronounced for the *Rbm20*^Δ*RRM*^ strips. For WT strips, slower myosin detachment at 2.2 μm sarcomere length effectively enhances cross-bridge contributions to thin-filament activation to augment tension production and Ca^2+^-sensitivity of the tension-pCa relationship. Previous studies have linked greater Ca^2+^-affinity of troponin C and greater opening of the N-terminus of troponin C with increases in strong cross-bridge binding (Hofmann and Fuchs, 1987; Wang and Fuchs, 1994; Terui et al., 2008, 2010; Smith et al., 2009; Li et al., 2014), and our current findings in WT strips and rat papillary muscle strips (Tanner et al., 2015; Pulcastro et al., 2016) imply this cooperative activation pathway becomes stronger at longer sarcomere lengths. The MgADP release rate (*k*_−*ADP*_) was 13% slower at 1.9 μm and 23% slower at 2.2 μm sarcomere length in *Rbm20*^Δ*RRM*^ strips, compared to WT strips, which would be expected to slow cross-bridge detachment and stabilize, or amplify thin-filament activation more greatly in *Rbm20*^Δ*RRM*^ strips. However, slower cross-bridge detachment rates did not enhance tension nor length-dependent activation of contraction in *Rbm20*^Δ*RRM*^ strips with greater titin compliance. Thus, cross-bridge contributions to thin-filament activation and increased Ca^2+^-affinity of troponin C may require titin interacting with the thin-filament or titin transmitting tension between the thick and thin-filament. Increased titin compliance in *Rbm20*^Δ*RRM*^ strips may compromise this titin interaction or tension transmission pathway, thereby depressing Ca^2+^-activated tension production and length-dependent activation of contraction.

Some muscle mechanics studies use large amplitude release-restretch protocols (~15% muscle length) to assess the cross-bridge rate of tension redevelopment (*k*_*tr*_), in comparison to the low amplitude strains used for stochastic length perturbation analysis (<0.15% muscle length). Skinned myocardial strips from WT and *Rbm20*^Δ*RRM*^ strips mice showed no differences in sarcomere length-dependent *k*_*tr*_ under maximally Ca^2+^-activated conditions (Methawasin et al., 2014). Previous studies using skinned myocardium from rats expressing the more compliant N2BA titin isoform have shown mixed reports of slower and faster *k*_*tr*_ values as sarcomere length increased (Patel et al., 2012; Hanft et al., 2014), compared to wild-type controls that predominantly express the stiffer N2B titin isoform. Herein we measured cross-bridge kinetics as [MgATP] varied, which allowed us to estimate cross-bridge rates of MgADP release (*k*_−*ADP*_) or MgATP binding (*k*_+*ATP*_; Table 2). As the rate of MgADP release limits cross-bridge detachment in a muscle fiber (Siemankowski et al., 1985), the ~12% slowing in *k*_−*ADP*_ from 1.9 to 2.2 μm sarcomere length drives the length-dependent slowing of cross-bridge detachment in WT strips. However, the length-dependent slowing of *k*_−*ADP*_ was nearly twice as great in *Rbm20*^Δ*RRM*^ strips (~22%) and *k*_−*ADP*_ was also slower at each sarcomere length when titin compliance increased. There was not a significant difference between cross-bridge MgATP binding rates at 1.9 vs. 2.2 μm sarcomere lengths for either genotype. These data support our previous observations that slowed MgADP release rate is the predominate step of the cross-bridge cycle that is responsible for the length-dependent slowing of cross-bridge kinetics (Tanner et al., 2015; Pulcastro et al., 2016). We do not think these slowed nucleotide handling kinetics in *Rbm20*^Δ*RRM*^ strips stem from any α-to-β myosin heavy chain isoform shift, because Methawasin et al. (2014) reported solely α-myosin heavy chain expression in both of these mouse lines. Moreover, these data also demonstrate that titin compliance influences sarcomere length-dependent cross-bridge nucleotide handling rates, and the effects of titin on cross-bridge kinetics become greater as sarcomere length increases.

Under relaxed conditions, both viscoelastic mechanical stiffness (Figure 2) and steady-state tension values (Table 1) were greater at longer sarcomere length, without any differences between the two genotypes. These differences stem from passive elements of the sarcomere being stretched or extended more greatly at 2.2 vs. 1.9 μm sarcomere length [i.e., titin and collagen (Granzier and Irving, 1995)]. We had anticipated that length-dependent increases in relaxed stiffness and tension would be greater for WT vs. *Rbm20*^Δ*RRM*^, similar to previous observations using skinned myocytes (Methawasin et al., 2014). However, Methawasin et al. (2014) also showed greater collagen expression in *Rbm20*^Δ*RRM*^ vs. heterozygous *Rbm20* knockout mice, which could be a compensatory mechanism to increase myocardial stiffness as titin compliance decreased in the homozygous mice. Given that skinned myocardial strips encompass some component of passive stiffness due to collagen that isn't present in isolated myocytes, it is possible that the mechanical characteristics of collagen, rather than titin, are dominating our relaxed muscle mechanics measurements. While previous studies suggested that greater passive tension values are correlated with greater Ca^2+^-activated tension production and length-dependent activation of contraction (Fukuda et al., 2001, 2003), our measurements do not support this mechanism driving length-dependent activation because relaxed stiffness and tension values were similar at each sarcomere length for both genotypes.

Thick-to-thin-filament spacing consistently decreases as sarcomere length increases in skinned and intact muscle preparations (Matsubara and Millman, 1974; Irving et al., 2000; Konhilas et al., 2002; Smith et al., 2009). Mechanical characteristics of titin influence this lattice spacing vs. sarcomere length relationship, showing that increased titin compliance can increase myofilament lattice spacing and affect the relationship between lattice spacing and sarcomere length (both increasing and decreasing the slope of this relationship; Cazorla et al., 2001; Fukuda et al., 2001, 2003, 2005; Irving et al., 2011). In addition, recent measurements show smaller myofilament lattice spacing values in *Rbm20* knockout rat myocardium at both short and long sarcomere length, compared to wild-type controls (Ait-Mou et al., 2016). Cross-bridge cycling rates have been shown to slow as thick-to-thin-filament spacing decreased in vertebrate and invertebrate muscle fibers that were osmotically compressed with Dextran (Krasner and Maughan, 1984; Kawai and Schulman, 1985; Smith et al., 2009; Tanner et al., 2012b) and with increases in sarcomere length in skinned (Adhikari and Wang, 2004; Tanner et al., 2015; Pulcastro et al., 2016), and intact (Milani-Nejad et al., 2013) cardiac muscle preparations. Therefore, increases in sarcomere length will accompany decreases in thick-to-thin-filament spacing, which could contribute to slower cross-bridge detachment at longer sarcomere length for both genotypes.

While reduced lattice spacing may slow cross-bridge cycling, this does not translate into increased length-dependent activation of contraction in *Rbm20*^Δ*RRM*^ strips (Hanft et al., 2014; Methawasin et al., 2014). Therefore, slowed cross-bridge cycling kinetics may not be the primary mechanism responsible for increasing Ca^2+^-activated tension at long sarcomere length (Patel et al., 2012), particularly when titin compliance increases from normal. Perhaps, titin interacts with the thin-filament to influence thin-filament activation and length-dependent activation of contraction, either directly or by influencing load (or strain) borne by thin-filament proteins (Terui et al., 2008; Hanft et al., 2014). This titin-thin-filament activation pathway may be suppressed with the more compliant titin in *Rbm20*^Δ*RRM*^ fibers, because titin is less taut and cannot effectively transmit tension between the M-band and Z-disks to maintain tension throughout the sarcomere. Thus, increases in cross-bridge duty ratio due to slowed detachment kinetics in *Rbm20*^Δ*RRM*^ fibers do not necessarily translate into the greater tension production due to a compromised capacity to generate tension or distribute tension throughout a more compliant sarcomere. Altogether, this would diminish ventricular function, and may scale with the expression ratio between the more compliant N2BA titin isoform and the stiffer N2B titin isoform. These impaired mechanisms of thin-filament activation and tension production may contribute to cardiac dysfunction and the associated cardiomyopathies in humans, rats, and mice bearing *RBM20* mutations that influence titin splicing (Makarenko et al., 2004; Nagueh et al., 2004; Guo et al., 2012; Methawasin et al., 2014).

## Author contributions

HP, PA, MM, and BT participated in performing the experiments and data collection. BT, WD, and HG conceived and designed the experiments. HP, PA, and BT analyzed the data. All authors helped interpret the data, write, and revise the manuscript, and have approved the final version of this manuscript.

### Conflict of interest statement

The authors declare that the research was conducted in the absence of any commercial or financial relationships that could be construed as a potential conflict of interest.
